# Ungulate Vehicle Collisions in a Peri-Urban Environment: Consequences of Transportation Infrastructures Planned Assuming the Absence of Ungulates

**DOI:** 10.1371/journal.pone.0107713

**Published:** 2014-09-24

**Authors:** Iñigo Zuberogoitia, Javier del Real, Juan José Torres, Luis Rodríguez, María Alonso, Jabi Zabala

**Affiliations:** 1 Estudios Medioambientales Icarus, S.L., Bilbao, Bizkaia, Spain; 2 Saitec S.A. Leioa, Bizkaia, Spain; 3 Arrigorriaga, Biscay, Spain; University of Sydney, Australia

## Abstract

Ungulate vehicle collisions (UVC) provoke serious damage, including human casualties, and a large number of measures have been developed around the world to avoid collisions. We analyse the main factors involved in UVC in a road network built in the absence of ungulates, where mitigation structures to avoid UVC were not adequately considered. Ungulate population greatly increased during the last two decades and now Roe Deer and Wild Boars are widely distributed over the study area, but even after this increase, the road network was not adapted to avoid UVC. A total of 235 Roe Deer (RDVC) and 153 Wild Boar vehicle collisions (WBVC) were recorded between January 2008 and December 2011. We randomly selected 289 sample points (87 RDVC, 60 WBVC and 142 controls) separated by at least 500 metres from the next closest point and measured 19 variables that could potentially influence the vehicle collisions. We detected variations in the frequency of RDVC on a monthly basis, and WBVC was higher at weekends but no significant differences were detected on a monthly basis. UVC were more likely to occur at locations where sinuosity of the road, velocity, surface of shrub and deciduous forest area were greater, the presence of fences entered with positive relationship and distance to the nearest building was less. RDVC were more likely to occur at locations where timber forest area increased and distance to the nearest building decreased and WBVC was related to open fields cover and also to the presence of fences. Sinuosity and velocity entered in both cases as significant factors. Major roads, in which the traffic volume is greater and faster, caused more accidents with ungulates than secondary roads. Nowadays, the high frequency of ungulate road-kills deserves a new strategy in order to adapt infrastructure and adopt mitigation measures.

## Introduction

Habitats and, consequently, wildlife populations are being increasingly fragmented due to increasing human population, urbanized areas, the extent of transport infrastructure, habitat transformation and agricultural intensification [1]. In this changing world, refuges free of perturbation are becoming scarce and wildlife is forced to live in highly human populated and changing habitats [2]. Interactions between large herbivores and transport infrastructures are expected to increase, since the road network and the traffic volumes are predicted to grow. Therefore, more populations will become susceptible to decline due to road mortality as the transportation infrastructure increases [3], [4]. Although most wild ungulate species are not threatened and are considered game species, they generate notable interest due to their size, medium-large animals, which can provoke serious damages, including human casualties [5]–[8]. Therefore, great efforts are being made to understand the causes of ungulate vehicle collisions (UVC) and to develop strategies to reduce them [11].

New transport infrastructures are normally planed and developed in natural environments, fragmenting habitats and wildlife home ranges. Environmental impact studies analyse the effect of transportation infrastructures on native wildlife populations, mainly ungulates and large carnivores, that already occupy the affected areas [7], [9]–[12]. However, to our knowledge, there are no documented analyses showing the effect of ungulate population recovery on a transportation infrastructure network which was developed in the absence of ungulates. In the 1980 s, there was a limited range of wild ungulates in northern Spain, Roe Deer (*Capreolus capreolus*) and Wild Boar (*Sus scrofa*) being restricted to small populations on the western border of the study area [13]. Since then, the road network has increased rapidly. But, as initially the hazards related to collision with large mammals were almost negligible, no fences, culverts and other mitigation structures were considered, apart from a few placed along the A8 and A68 highways. In fact, mitigation measures to prevent road-kills were partly taken into account in some new roads since 2008, following the increasing interest to deal with this problem [14]. During this 30 year period, ungulate collisions just started to become a threat whilst the meso-carnivore road-kills were constant [15]. In fact, this emerged as the main non-natural cause of mortality of some endangered carnivores [16]. However, due to the small size of the affected animals, this was not significant in terms of vehicle damage or human injuries and authorities did not consider it as a problem. Meanwhile, in common with other areas of the Iberian Peninsula [17], the population and the distribution area of wild ungulates increased rapidly, spreading throughout the country [18]. In the first decade of this Century, road-kills, agricultural damage and the numbers of game hunted continuously increased [19], and now the wild ungulate populations are dense and widely distributed. Nevertheless, the road network did not evolve in such a way as to avoid UVC, and now the high frequency of ungulate road-kills deserves a strategy in order to adapt infrastructure and adopt mitigation measures.

The main goal of this paper, therefore, is to identify the main factors involved in UVC, the road stretches where the UVC risk is higher than others and where mitigation measures should be adopted to reduce the problem.

### Study area

The study area covered the whole administrative area of Bizkaia (Basque Country, northern Spain; surface = 2,384 km^2^; coordinates from 43°11’00’’ to 43°12’70’’N and from 3°12’70’’ to 2°13’10’’W). Here, human density is amongst the highest in Western Europe (523.6 inhabitants/km^2^, [20]). The territory is hilly and characterised by the presence of extensive urban and industrialised areas connected by 1,317.1 km of roads, of which 203.5 km are highways. Highways are fenced to keep wildlife out of the road lanes, although fences are not equally distributed and some stretches show unmaintained fencing with holes made by wildlife. The A8 and A68 highways mostly have 2 m high fencing whilst more recently built highways provide 1.5 m high fencing, that in every case were installed during the road building. The average traffic density is 9,837 vehicles per day and 4,704.9 millions of vehicles per km/year, of which 64.5% belong to the highway network [21].

More than 50% of the area is dedicated to forestry, at the expense of traditional small-scale farming. Most of the wood produced comes from plantations of *Pinus radiata* and *Eucalyptus globulus, E. camaldulensis* and *E. nitens*, while the traditional patchwork of woodland, pasture and small-holdings has been greatly reduced during recent decades [22].

## Materials and Methods

### Ungulate vehicle collision (UVC)

Data on UVC were obtained from the road accident statistics of the “Departamento de Obras Públicas y Transportes de la Diputación Foral de Bizkaia” which is the main Road Authority in Bizkaia. Overall 235 Roe Deer and 153 Wild Boar vehicle collisions were recorded between January 2008 and December 2011. Each record contained information about species, place and date. The location of each collision was identified by the kilometric point of the road (data recorded in stretches of 100 m ). Accuracy of the UVC location is ±50 m. We used a dataset (Database S1) from a geographical information system. The software used was Quantum GIS 1.7.4.

### Sampling point selection

**Figure 1:**
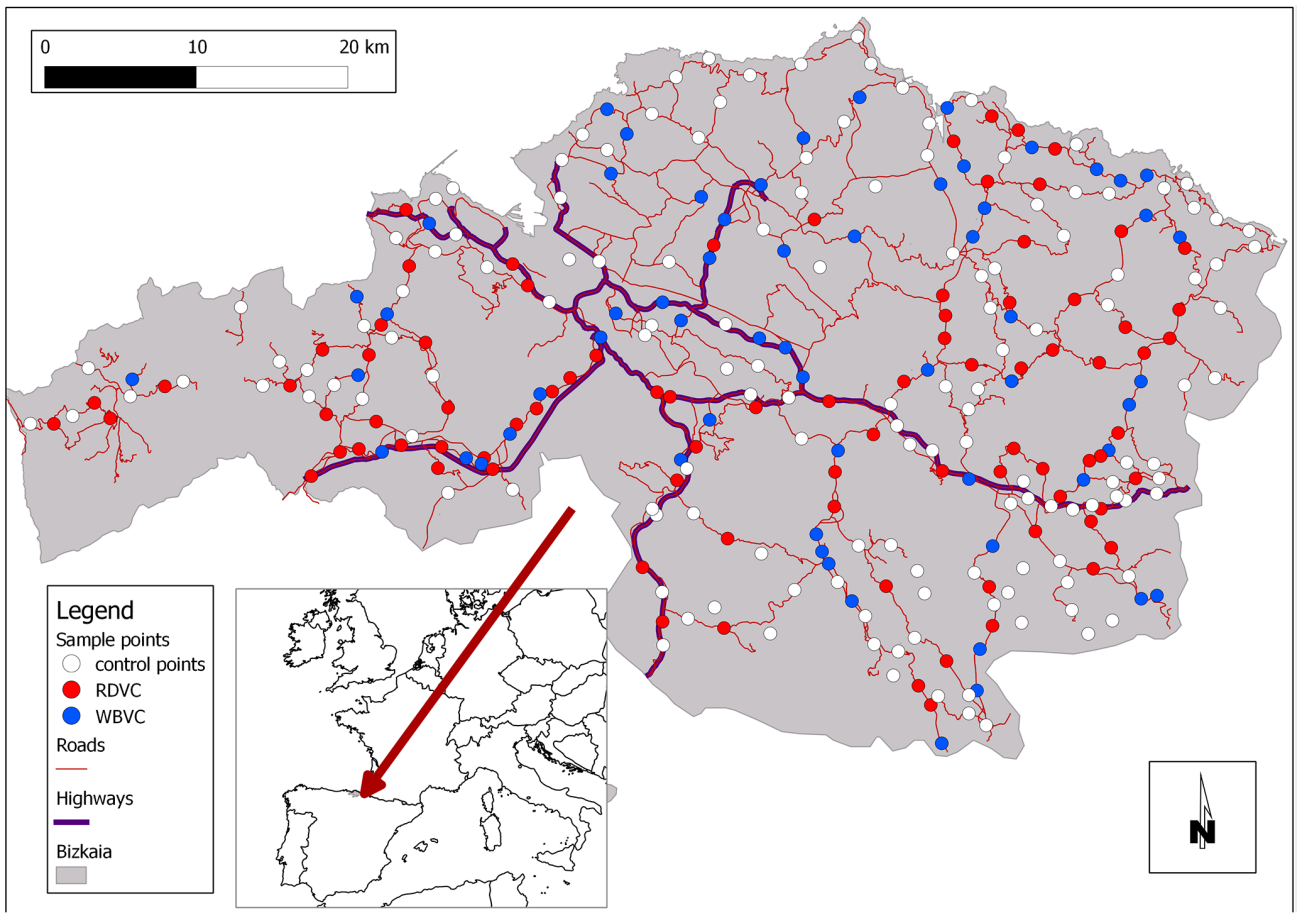
Sampling point distribution. Map showing the distribution of the sample points randomly selected for the Generalized Lineal Model (GLM) analysis: 87 Roe Deer vehicle collisions (RDVC, red dots), 60 Wild Boar vehicle collisions (WBVC, blue dots) and 142 non-accident control points (white dots) on the roads and highways in the study area, Bizkaia, Northern Iberian Peninsula, between 2008 and 2011.

In order to avoid pseudo-replication of the sampling site factors, we randomly selected 289 sample points separated by at least 500 m from the nearest point: 87 Roe Deer vehicle collision (RDVC) points, 60 Wild Boar vehicle collision (WBVC) points and 142 non-accident control points in which no collision had been detected during the study period (Fig. 1).

### Landscape and traffic factors

Landscape data were obtained from a combination of digital land-use maps, topographic maps and satellite imagery at a scale of 1∶5,000, available as free SIG data in the Basque Government website [23]. Traffic data were obtained from the annual reports of the “Departamento de Obras Publicas y Transportes de la Diputación Foral de Bizkaia” [24], in particular, there is a wide network of sampling stations for traffic monitoring for which it is possible to download the traffic volume of every road [21]. Average vehicle speed was obtained from the traffic sampling stations, but in those few cases in which data were not available we considered the speed limits. The presence of fences was also checked in the field and was contrasted using the Google Street View Programme [25].

We generated three categories of variables (habitat structure, topographic factors and road factors, Table 1) of biological relevance to wild ungulates [17], [26]. In total, we selected 19 variables that could potentially influence the UVC (Table 1). We created a buffer area of 500 m radius for every sampling point in which we measured the surface covered by each of the habitat variables, and a buffer area of 100 m radius to calculate the slope.

**Table 1:** Environmental parameters, measured within 500-m radius buffers, surrounding ungulate vehicle collision (UVC) and control sites in the model.

Variables/codes	Description
**Habitat structure**	
DECD	Deciduous forest surface area (m^2^)
HOAK	Holm Oak forest surface area (m^2^)
TIMB	Exotic timber plantation surface area (m^2^)
SHBR	Shrub cover surface area (m^2^)
OPEN	Open grass fields and agricultural surface area (m^2^)
FRAG	The number of vegetation patches
AFRAG	The average surface of vegetation patches (m^2^)
DRIV	Distance to the nearest river (m)
DFOR	Distance to the nearest forested patch (m)
DBFOR	Distance between two forested patches placed in each side of the road (m)
DBUIL	Distance to the nearest building (m)
DPOP	Distance to the nearest population nuclei (>5 buildings) (m)
**Topographic factors**	
ALT	Altitude of the sampling point (m a.s.l.)
SIN	Road sinuosity. The relationship between the actual length of the road and the Euclidean distance between each end of the road in the 500 m radius buffer area. Larger values of sinuosity indicate more curves in the road.
SLOP	Slope. The number of 20 m contour lines which cross a circle of 100 m radius around the sampling point
**Road factors**	
VEL	Average velocity of vehicles in each sampling point (km/h)
TRAF	Traffic volume. Number of vehicles per day
TRAFH	Traffic volume of heavy vehicles per day
FENCE	Presence of fence (categorical variable)

### Statistical Analysis

The number of potential habitat variables was reduced by evaluating correlation and fit across spatial scales. Habitat variables were assessed for multicollinearity (r >0.65) using Pearson’s correlation matrix. Hence, the variables TRAFH, FRAG, DPOP and DBFOR were excluded from the analysis, (see Table 1).

Weekly and monthly frequencies of vehicle collisions were analysed using the Chi Square test.

Univariate tests for differences in the environmental and road traffic factors between collision and non-collision sites were computed using non-parametric Mann-Whitney’s test [27]. Firstly, we compared variables between non-collision sites and UVC sites, and secondly, we divided ungulates into Roe Deer and Wild Boar and developed univariate analysis comparing each one with non-collision sites and between them. Latterly, we ran a multivariate non-parametric test for differences amongst the three groups (non-collision, RDVC and WBVC sites) using Kruskal-Wallis test.

Categorical variables were tested using χ^2^ function. Significance level was set at *P*<0.05.

We used Monte Carlo simulations to evaluate the probability that observed differences in velocity and traffic volume between RDVC, WBVC and control sites could have occurred by chance. These variables were the only significant factors shared in both cases: RDVC and WBVC (see in results). This method has several advantages since it allows frequency distributions to be generated and hypothesis testing based on real field data and not theoretical distributions (i.e. [28], [29]). Firstly, we resampled an equal number of observed data (VEL and TRAF) for the three groups using the “shuffle” function implemented in PopTools version 3.2.5 for Excel [30]. This procedure was repeated 1000 times in each case. The mean value obtained in the resample of each case was used to run the Monte Carlo simulations, using the simulation tool: Monte Carlo Analysis function. Three data matrixes were obtained with the mean values of every 1000 resampling, displayed in a histogram and a mathematic function. Secondly, critical values of significance were generated by counting the number of randomization cases (for the control sites) that resulted in an equal or larger/smaller value than the observed median values of RDVC and WBVC divided by the number of randomizations [31]. Tests were two-tailed and significance level was set at α = 0.05.

Finally, we used generalized linear models (GLM) analysis, with collision/non collision as a binary response variable with a logit link function, to identify which environmental and road related variables best predicted the likelihood of casualties for Roe Deer, Wild Boar and for both [32]. We included the RDVC, WBVC and control sites into 500 m radius circles (n = 289). Each case was coded either 1 (with collision) or 0 (non-collision) and described in terms of fifteen explanatory variables (the 19 variables in Table 1 except to the four auto-correlated factors) obtained from field measurements or from a geographic information system. Three different analyses were run considering both ungulates together (UVC), or only RDVC and WBVC. We examined the ability of each variable to predict road mortality using univariate logistic regression. We selected only those variables for the model when the significance of the Wald test was <0.1. Next, we used stepwise forward regression procedures to build the models, leaving out 20% of the original data for cross-validation procedures in order to evaluate the explanatory power of each model. To select the model best supported by our data, Akaike’s Information Criterion, corrected for small sample size (AIC_c_) was used. We calculated AIC_c_ differences between candidate models and selected as best the one showing the lowest AIC_c_ value_._ Models within 2 units of ΔAIC_c_ were considered to have substantial empirical support, models with ΔAIC_c_ 4–7 were considered to have substantially less empirical support, whereas models with more than 10 units of ΔAIC_c_ were considered to have essentially no support from the data. We also calculated the Akaike Weights (wAIC_c_) (i.e. the weight of evidence in favour of that model being the best in the set, [33]) among candidate models [32].

Statistical analyses were performed with SPSS v18 (SPSS INC., Chicago, IL, U.S.A.).

## Results

The frequency of RDVC did not vary with the days of the week (*χ* ^2^_6_ = 11.609, *P* = 0.07), but there were significant differences between months, April and May followed by July and August being the months with highest RDVC (*χ* ^2^_11_ = 64.092, *P*<0.001). The frequency of WBVC was higher on weekends followed by Wednesdays (*χ* ^2^_6_ = 14.769, *P* = 0.022), but there were no significant differences between months (*χ* ^2^_11_ = 9.588, *P* = 0.567) although a weak peak was observed in autumn.

In a first approximation, running a descriptive analysis in order to compare UVC with control sites, three road factors (SIN, TRAF and VEL) and one habitat variable (SHBR) showed statistically significant differences (Table 2). The roads with more curves prompted fewer collisions than straight roads and the likelihood of collisions increased with the traffic volume. The area of shrub was the only significant habitat variable for wild ungulates, collisions being higher in those areas dominated by this type of vegetation.

**Table 2:** Mean, standard deviation and range (brackets) of the environmental and road variables of 147 ungulate vehicle collisions (UVC) localities and 142 non-collision control sites.

Variables	UVC (n = 147)	Control (n = 142	U Mann-Whitney
**DECD** (m^2^)	60003±70267	55130±73565	6534.5
	(631–356006)	(14–508430)	
**HOAK** (m^2^)	12723±41636	10053±40308	10287
	(0–283668)	(0–349371)	
**TIMBER** (m^2^)	325580±195471	322478±230581	10205
	(0–752813)	(0–769761)	
**SHBR** (m^2^)	50890±93760	27854±53512	9037 (*)
	(0–544428)	(0–261814)	
**OPEN** (m^2^)	329670±191652	358419±231107	9809
	(0–772543)	(0–772542)	
**AFRAG** (m^2^)	97210±50139	104420±58577	9524
	(32189–257514)	(32189–386271)	
**DRIV** (m)	188±224	237±310	9715.5
	(3–1509)	(5–2500)	
**DFOR** (m)	55±124	88±150	9976
	(3–1293)	(2–997)	
**DBUIL** (m)	149±178	203±298	10291
	(3–1419)	(3–1731)	
**ALT** (m a.s.l.)	178±147	200±154	9449.5
	(10–680)	(10–720)	
**SIN**	1.13±0.20	1.14±0.16	9025.5 (*)
	(1–2.1)	(1–1.7)	
**SLOP**	2.9±1.5	2.8±1.5	10221
	(0–7)	(0–6)	
**VEL** (km/h)	73.9±19.0	61.0±24.6	6255 (**)
	(30–120)	(20–120)	
**TRAF** (veh/day)	11682±17665	9096±18955	7534.5 (**)
	(191–119113)	(25–136190)	

Significance of the Mann-Whitney test is indicated with asterisks (* *P*<0.05, ** *P*<0.01).

RDVC sites were characterized by higher traffic volume and velocity compared with control sites (Table 3). Traffic volume and velocity also influenced WBVC, although WBVC sites were also characterized by lower altitude and road sinuosity than control sites (Table 3).

**Table 3:** Mean, standard deviation and range (between brackets) of the environmental and road variables of 87 Roe Deer vehicle collisions (RDVC) and 60 Wild Boar vehicle collisions (WBVC) localities.

	RDVC (n = 87)					WBVC (n = 60)				
	Mean		SD	Range	U	Mean		SD	Range	U
**DECD** (m^2^)	59289	±	73911	(631–356006)	4052	61096	±	65026	(2076–325041)	2482
**HOAK** (m^2^)	10470	±	38918	(0–245449)	6102	15990	±	45428	(0–283668)	4035
**TIMBER** (m^2^)	347995	±	195452	(0–692298)	5668	293078	±	192490	(0–752813)	3983
**SHBR** (m^2^)	45987	±	85489	(0–462583)	5440	58000	±	104948	(0–544428)	3597
**OPEN** (m^2^)	314278	±	179345	(0–772543)	5592	351989	±	207731	(2970–756319)	4217
**AFRAG** (m^2^)	97088	±	45137	(32189–257514)	5798	97388	±	57019	(34847–257514)	3725
**DRIV** (m)	175	±	189	(3–949)	5654	206	±	267	(5–1509)	4061
**DFOR** (m)	58	±	151	(3–1293)	5721	52	±	71	(3–330)	4255
**DBUIL** (m)	145	±	143	(3–718)	6117	156	±	221	(3–1419)	4174
**ALT** (m a.s.l.)	191	±	151	(20–680)	5946	160	±	142	(10–640)	3504*
**SIN**	1.1	±	0.2	(1–2.1)	5611	1.1	±	0.2	(1–1.7)	3414*
**SLOP**	2.9	±	1.5	(0–7)	6128	3.0	±	1.6	(0–7)	4093
**VEL** (km/h)	70.0	±	17.5	(30–109)	4191**	79.6	±	19.9	(30–120)	2064**
**TRAF** (veh/day)	10111	±	16156	(295–98780)	4873**	13960	±	19566	(191–119113)	2622**

Results of Mann-Whitney’s U test for the comparisons between RDVC and WBVC with control sites. Significance levels: * *P*<0.05, ** *P*<0.01.

**Figure 2:**
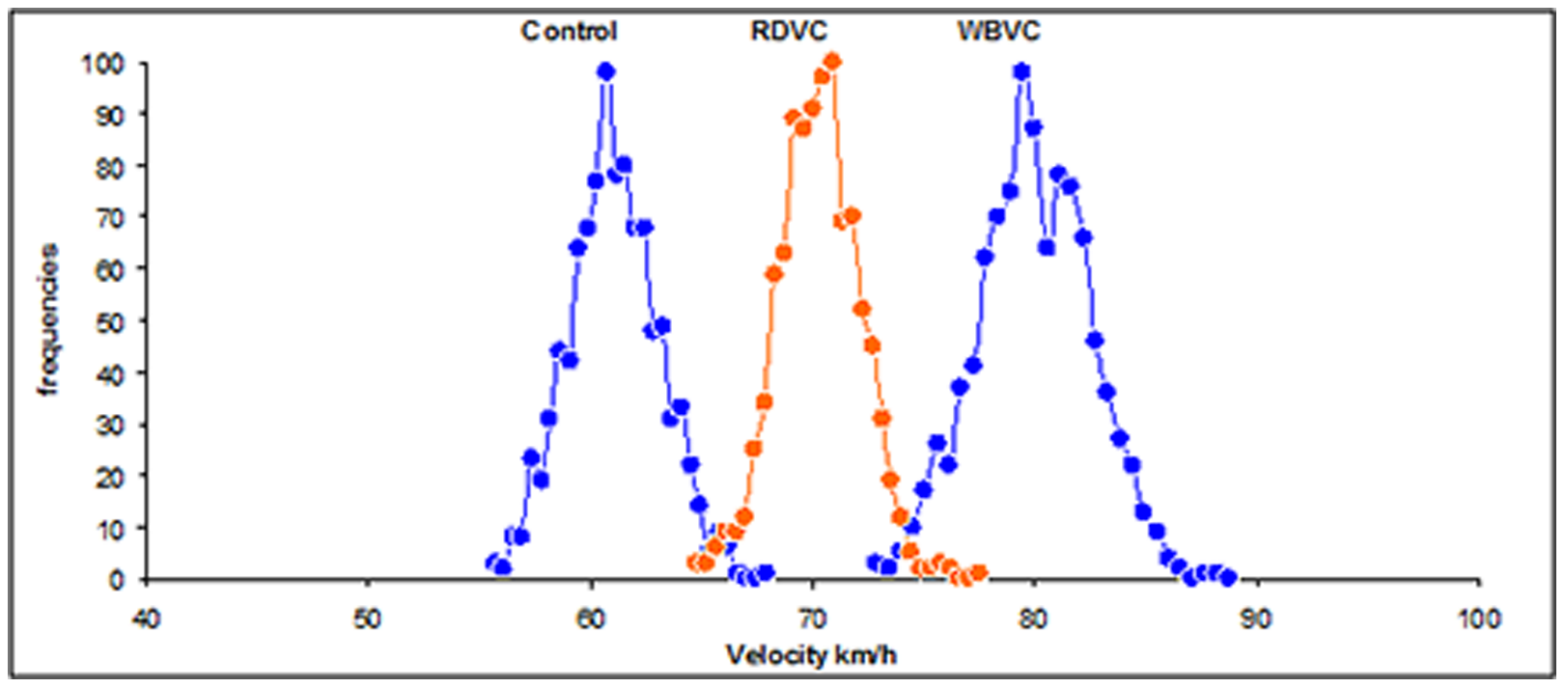
Mean velocities. Mean values of velocity (km/h) for non-collision control sites, Roe Deer vehicle collisions (RDVC) and Wild Boar vehicle collisions (WBVC) of the resample (1000 times) after Monte Carlo simulations.

**Figure 3:**
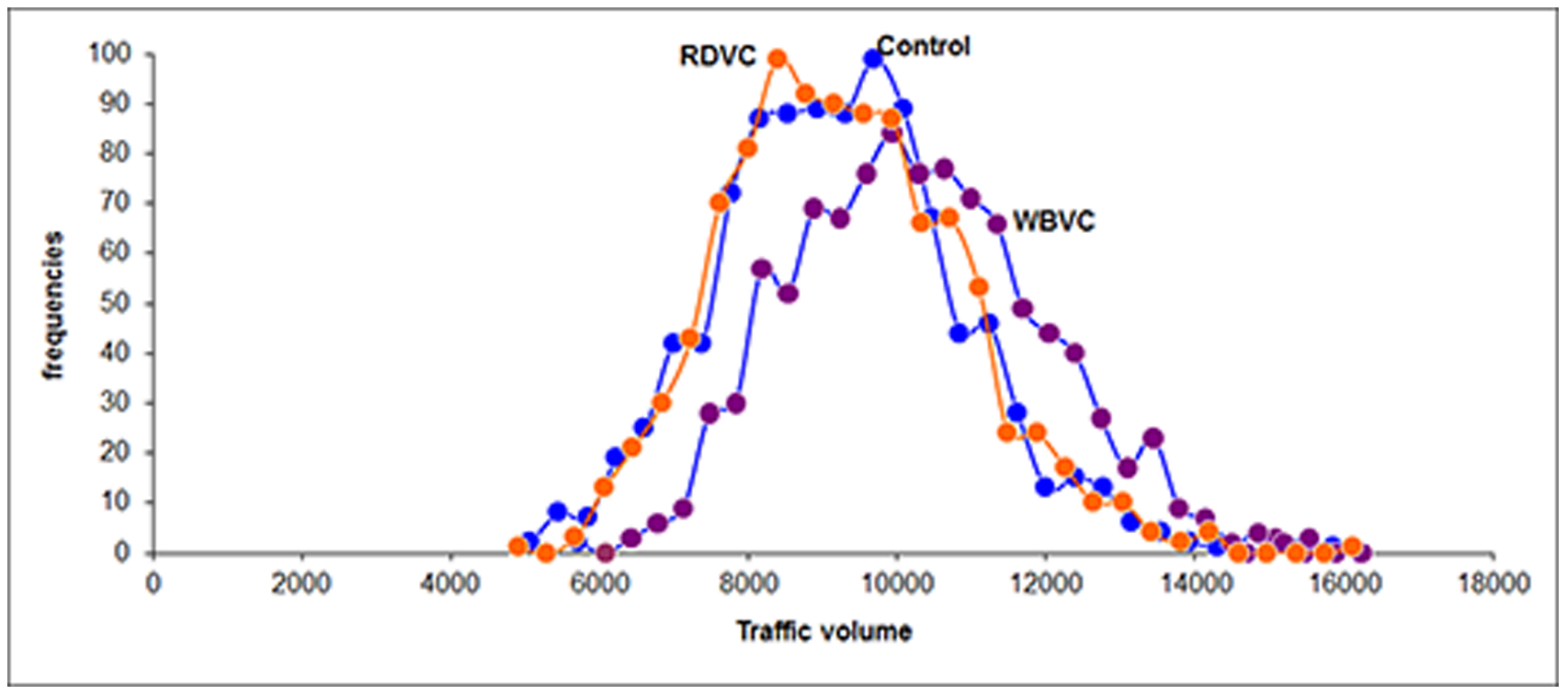
Mean traffic volume. Mean values of traffic volume (TRAF, vehicles/day) for non-collision control sites, Roe Deer vehicle collisions (RDVC) and Wild Boar vehicle collisions (WBVC) of the resample (1000 times) after Monte Carlo simulations.

Only velocity (Kruskal-Wallis Test, *H* = 41.39, *P*<0.001) and traffic volume (Kruskal-Wallis Test, *H* = 19.39, *P*<0.001) showed significant differences when comparing control, RDVC and WBVC sites. When running Monte Carlo simulations, the analysis confirmed significant differences between the three groups (*P*<0.001), in which WBVC were detected at higher speed than RDVC and RDVC at higher speeds than control sites (Fig. 2). But, this was not the case for traffic volume, since no differences were detected between the groups (*P*>0.05, Fig. 3). Hence, major roads, in which the traffic volume is greater and its speed is higher, caused more accidents with ungulates than secondary roads, although those highways with a very high traffic volume showed fewer collisions than expected.

Regarding the presence of fences, there was not the expected negative relationship between collisions and fences, neither for Roe Deer nor for Wild Boar (Table 4).

**Table 4:** Number of cases of control sites, ungulate vehicle collisions (UVC), Roe Deer vehicle collisions (RDVC) and Wild Boar vehicle collisions (WBVC) depending on the absence or presence of fences and the statistical value of the Chi square test.

Fence	Control	UVC	RDVC	WBVC
**No**	126	130	79	51
**Yes**	16	17	8	9
***χ*** **^2^**		0.006	0.246	0.542
***P***		0.936	0.620	0.462

Results of the GLM predicted that UVC were more likely to occur at locations where the sinuosity, velocity, the amount of shrub cover and deciduous forest were greater, the presence of fences gave a positive relationship and distance to the nearest building was less (Table 5 and 6). Moreover, if we consider the models within 2 units of ΔAIC_c_ of the most parsimonious model, the presence of timber forest would be included as another determinant factor that positively affects UVC (Table 5).

**Table 5:** Candidate road and landscape based models predicting ungulate vehicle collisions (UVC), Roe Deer vehicle collisions (RDVC) and Wild Boar vehicle collisions (WBVC) in the study area, Bizkaia, from 2008 to 2011.

Ranking	Model	n	k	AIC*_c_*	ΔAIC_c_	AIC*_c_* w_i_
**UVC**						
1	SIN+VEL+SHRB+FENCE+DBUIL+DECD	223	6	303.52	0	0.269
2	SIN+VEL+SHRB+FENCE+DBUIL	223	5	303.76	0.24	0.238
3	SIN+VEL+SHRB+FENCE	223	4	304.64	1.12	0.154
4	SIN+VEL+SHRB+FENCE+DBUIL+DECD+TIMB	223	7	304.47	0.95	0.167
5	SIN+VEL+SHRB+FENCE+DBUIL+DECD+TIMB+HOAK	223	8	306.39	2.87	0.064
6	SIN+VEL+SHRB+FENCE+DBUIL+DECD+TIMB+HOAK+OPEN	223	9	306.35	2.83	0.065
7	SIN+VEL+SHRB+FENCE+DBUIL+DECD+TIMB+HOAK+OPEN+TRAF	223	10	308.05	4.53	0.028
8	All variables	223	15	318.81	15.29	0.001
**RDVC**						
1	TIMB+DBUIL	223	2	297.16	0	0.285
2	TIMB+DBUIL+SIN+VEL	223	4	297.99	0.8	0.189
3	TIMB+DBUIL+SIN	223	3	297.69	0.5	0.220
4	TIMB+DBUIL+SIN+VEL+DECD	223	5	298.99	1.8	0.115
5	TIMB+DBUIL+SIN+VEL+DECD +SHBR	223	6	299.99	2.8	0.070
6	TIMB+DBUIL+SIN+VEL+DECD +SHBR+SLOP	223	7	300.47	3.28	0.055
7	TIMB+DBUIL+SIN+VEL+DECD +SHBR+SLOP+FENCE	223	8	301.19	4.00	0.038
8	TIMB+DBUIL+SIN+VEL+DECD +SHBR+SLOP+FENCE+DRIV	223	9	302.74	5.55	0.018
9	All variables	223	15	315.01	17.82	0.000
**WBVC**						
1	VEL+FENCE	223	2	224.35	0	0.271
2	VEL+FENCE+SLOP	223	3	225.09	0.74	0.186
3	VEL+FENCE+SLOP+OPEN	223	4	225.40	1.05	0.159
4	VEL+FENCE+SLOP+OPEN+SHRB	223	5	225.49	1.14	0.153
5	VEL+FENCE+SLOP+OPEN+SHRB+DECD	223	6	226.78	2.43	0.080
6	VEL+FENCE+SLOP+OPEN+SHRB+DECD+HOAK+TIMBER	223	8	227.12	2.77	0.068
7	VEL+FENCE+SLOP+OPEN+SHRB+DECD+HOAK	223	7	228.10	3.75	0.041
8	VEL+FENCE+SLOP+OPEN+SHRB+DECD+HOAK+ALT	223	9	229.01	4.66	0.026
9	All variables	223	15	242.10	17.75	0.000

Models estimated and ranked by Akaike Information Criteria (AICc). K is the number of estimable parameters, ΔAIC_c_ shows the difference between the top model and each candidate model, and AIC*_c_* w**_i_** is the relative weighting of that model.

**Table 6:** Coefficients of the three most parsimonious GLM models describing the influence of environmental and road traffic factors on the probability of ungulate vehicle collisions (UVC), Roe Deer vehicle collisions (RDVC) and Wild Boar vehicle collisions (WBVC) on the roads of the study area between 2008–2011.

	β Coefficient	*S.E.*	*χ* ^2^ Wald	*P*
**Model for UVC**				
Constant	7.608	1.6941	20.170	0.000
FENCE	1.971	0.5988	10.833	0.001
SIN	2.074	0.8602	5.813	0.016
VEL	0.051	0.0097	27.449	0.000
DECD	3.25E-6	2.122E-6	2.348	0.125
SHBR	5.001E-6	2.312E-6	4.678	0.031
DBUIL	-0.001	0.0007	2.839	0.092
**Model for RDVC**				
Constant	1.061	0.2766	14.719	0.000
TIMB	1.696E-6	7.951E-7	4.355	0.037
DBUIL	−0.002	0.0008	3.811	0.051
**Model for WBVC**				
Constant	5.949	1.2227	23.673	0.000
FENCE	1.513	0.6452	5.495	0.001
VEL	0.046	0.0102	19.968	0.000

If we consider specific results, the most parsimonious model showed that RDVC were more likely to occur at locations where the amount of timber forest surface increased and distance to the nearest building decreased (Table 5 and 6). Although, if we consider the two models within 2 units of ΔAIC_c_, road sinuosity and velocity would also be linked as two other variables affecting the probability of collision with Roe Deer.

Finally, results showed that velocity and fences, both with positive values were the main factors which would determine the likelihood of collision with Wild Boars (Table 5 and 6). In this case, considering the ΔAIC_c_, three models within the 2 units could be also considered, which included slope, sinuosity and the surface of open fields respectively.

## Discussion

The temporal distribution of ungulate-vehicle collisions was not random. According to previous studies (reviewed in [5]), Roe Deer showed two peaks in vehicle collisions, in spring and during the rutting season, mainly in July. Yearlings disperse during late winter or spring, males dispersing further than females, increasing the vehicle collision risk until they finally arrive at their permanent settlement in the summer new area [34], [35]. Later, in summer, during the rutting season, territorial Roe Deer activity and movements increase which is likely to enhance opportunities for mating [36]. Moreover, the size of the home range of breeding females increases with increasing reproductive success, tending to use larger areas in April, July and August in order to adjust their home range size to the amount of resource they can obtain [37]. Out of these periods, the incidence is lower due, among other causes, to the strong territorial behaviour of Roe Deer and the relative small home ranges, including short movements between the foraging areas and the resting sites [38].

In the case of Wild Boars, although no significant differences were found in the collision frequency between months, results showed a slight peak in autumn, which is in accordance with previous studies [5], [39]. This peak is related to the game hunting activity that drives individuals out of the habitual resting sites, increasing home ranges [40]–[43]. Moreover, results showed an increase of accidents during weekends, when more traffic density is observed [21] and game hunting activity is allowed.

UVC were strongly influenced by the traffic speed and the road sinuosity, and both factors were negatively correlated (R = −0.51, *P*<0.001). The traffic speed is higher on straight roads and vehicles must reduce velocity depending on the number and typology of curves on a road. Therefore, ungulates were more susceptible to collision on straight stretches, mainly in highways where velocity is 20–40 km/h higher than on conventional roads.

**Figure 4:**
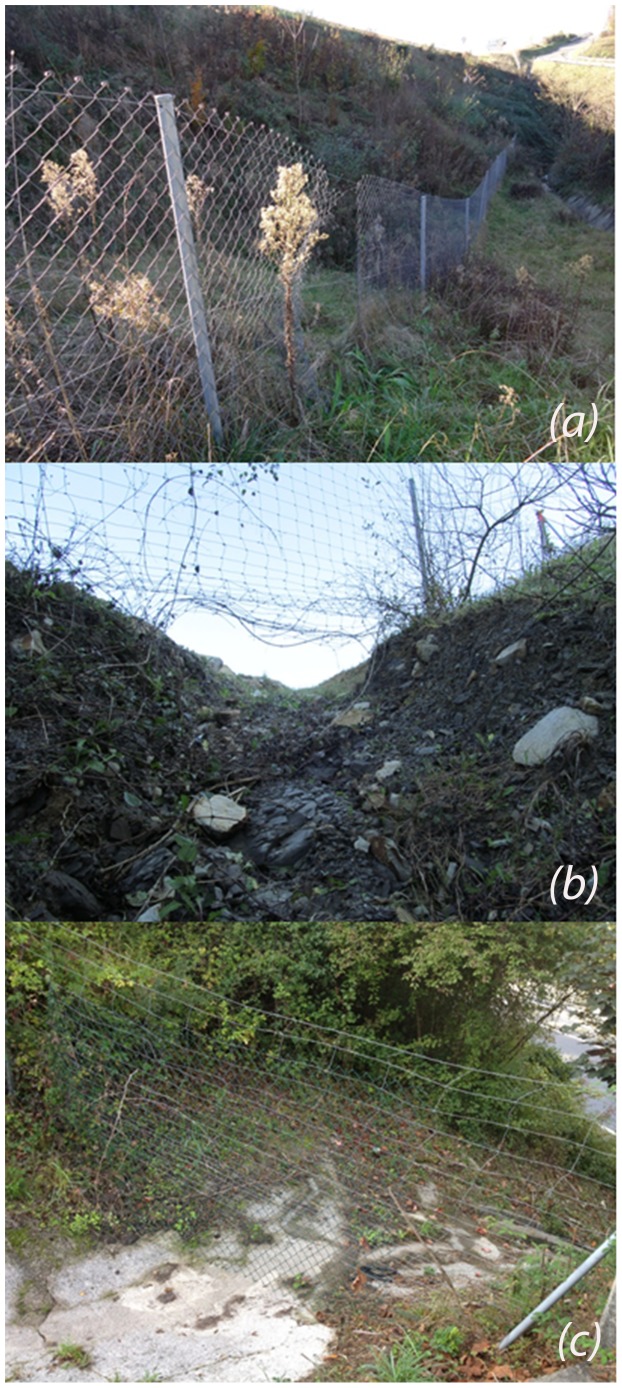
Poor fencing detected in the highways of the study area. Examples include: openings in fences (a), stretches inadequately buried into the ground (b) and damaged stretches in which agile ungulates may jump over the fence (c).

Contrary to expectations, fences did not significantly reduce UVC, in fact, the presence of fences increased Wild Boar collisions with vehicles. These results suggest a lack of efficiency on of fences in the study area, as the correct planning of fence and wildlife passages notably reduces road mortality of large mammals [44]. Two separate but not exclusive hypotheses are considered to explain this problem: the characteristics of the fences and the lack of wildlife passages. Five highway stretches were constructed from 1990 to 2008, when the wild ungulate population had already started to increase and disperse but they were not one of the main considerations at the time. The fences of these stretches (1.5 m in height at both sides of the highway) were adapted to prevent medium sized mammals entering the road, but wildlife passages were not taken into account or, in the latter cases, were poorly developed. Therefore, the new stretches acted as a barrier to medium and large mammal movements, forcing them to find different ways to cross fences: (1) through openings in fences, (2) in areas where the fence was inadequately buried into the ground or (3) in some areas in which the topography of the terrain let agile ungulates, mainly Roe Deer, jump over the fence of 1.5 m height (Fig. 4) [45]. Moreover, deer are intelligent animals that learn from observing others and once they penetrate the fence they can repeatedly use the same path [45]. The problem arises once inside, when animals cannot find the exit from the opposite side and they display nervous behaviour and run along the road. In such occasions poorly erected or designed fences may act as traps for animals and cause accidents instead of avoiding them. Poorly designed fences are believed to be the cause for Roe Deer and Wild Boars appearing inside cities, close to the exits of highways. This could be prevented by installing one-way exit structures, allowing ungulates to cross fences outwards from the road.

The observed differences in the traffic velocity between Roe Deer and Wild Boars could be related to the differential behaviour. RDVC were mainly produced in relatively low traffic velocity roads (on average of 70 km/h), whilst Wild Boars were killed in 10 km/h faster roads (on average of 80 km/h Fig. 2). Roe Deer are more nervous and unpredictable than Wild Boars (I. Zuberogoitia, personal observations). Roe Deer appear suddenly in the road, sometimes running or jumping, surprising drivers even at low speed. However, Wild Boar are relatively more predictable and quiet and drivers can easily detect them, reducing speed and avoiding collision, except in those cases in which speed is high and the reaction time is insufficient to avoid collision.

High traffic roads may act as movement barriers to ungulates. We found that Roe Deer and Wild Boar collisions occur in roads with an average of 10,000 and 14,000 vehicles per day respectively, and the likelihood of collisions decreased as the traffic volume increased (Fig. 3). Our results are consistent with previous studies showing that traffic density does not affect UVC in a linear relationship [36], [40], [43]. Furthermore, Madsen et al. [36] found Roe Deer can keep home ranges close to high traffic density roads and never cross them, suggesting that high traffic volume may constitute home-range boundaries for the species. In fact, high traffic density roads have been suggested to result in movement barrier for ungulates in previous studies [8], [39].

In addition to higher traffic volumes and velocity, Roe Deer collision risks were also higher close to human settlements, and areas with greater amount of timber harvest than control sites. Higher RDVC near human settlements is contrary to other neighbouring regions [7], likely due to the high human density of the study area and the nervous and impatient behaviour of Roe Deer when they have to pass close to inhabited buildings. Contrasting with the natural behaviour when Roe Deer cross roads far from inhabited areas which allows them to pay more attention to vehicles. The observation that RDVC were closely related to the timber plantation surface area are in agreement with previous studies of deer in urban areas of Canada [46] and Roe Deer in Germany [26]. Roe Deer may find appropriate resting sites and natural corridors to move within pine and eucalyptus forest, which most of the time are occupied by dense shrub stratus (see [47]). However, herbaceous biomass is scarce in this type of habitat and Roe Deer must move to open habitats or openings in the deciduous forests to forage, thereby crossing roads and increasing the odds of being killed [48]. With this argument, Madsen *et al*. [36] suggest that dense vegetation close to roads would increase the RDVC risk, although we did not detect any relationship between distances to the forest edges and RDVC. We suspect that this null effect could be due to the accuracy of our collision data (±50 m) and the changing habitats found in the study area. Areas where forest with dense undergrowth are close to roads could be fenced, guiding Roe Deer to areas where speed can be regulated to minimize collision risk, particularly in high Roe Deer density areas.

In the case of Wild Boars, the slope and the grass field surfaces entered into the model with velocity and sinuosity. Many roads pass between narrow hillsides, where Wild Boars use roadsides in order to look for adequate passages. The straight stretches between curves, where vehicles increase velocity, were the most dangerous. Wild Boars rest in forests and shrub areas and although they can forage inside them, they frequently move to open areas (grass fields and agricultural lands) at night, where they easily find herbaceous roots and invertebrates [43], [49], [50]. In our case, road side shrub cover increased the chance of collision. Road side vegetation management can provide a short to medium-term option to manage risks and guide animals to safe crossing areas.

## Conclusions

The results of our models mostly agree with previous research when determining the key predictor variables for ungulate collision, except for the scarce and contradictory effect of the fences for reducing kills due, among others, to the absence of adequate wildlife passages. The design of the current road infrastructure of Biscay is out-dated with regard to wildlife conservation, and has demonstrated adverse effects for wildlife and road users. On one hand, this road network has been significantly affecting mammals and other vertebrates for many years [47] and, only recently, authorities have started to seriously consider habitat fragmentation [51], [52]. On the other hand, the alarming increase of UVC in recent years and the consequences related to this have raised awareness regarding inadequate planning, and emphasis on the need to make improvements to road infrastructures so that they conform to modern conservation and safety strategies. In order to achieve this, a large number of costly changes need to be carried out. Wildlife passages, in combination with fencing, are required to reduce habitat fragmentation and road mortality. Notwithstanding, the cost of maintaining the current situation would be much higher [53]. To this purpose, our results provide useful information to help reduce collisions in the short to medium term by managing vegetation to reduce ungulate activity in the vicinity of areas of conflict, to improve the efficiency of fences, to reduce vehicle speed in dangerous stretches and to place passages in the areas where they are best suited and manage surrounding vegetation.

## Supporting Information

Database S1 **Original data set.**
(XLSX)

## References

[1] Prugh LR, Hodges KE, Sinclair ARE, Brashares JS (2008) Effect of habitat area and isolation on fragmented animal populations. Proc Natl Acad Sci 105: 20770–20775. 19073931 10.1073/pnas.0806080105 PMC2634894

[2] Andersen R, Linnell JDC, Langvatn R (1996) Short-term behavioural and physiological response of moose *Alces alces* to military disturbance in Norway. Biol Conserv 77: 169–176.

[3] Forman R, Sperling D, Bissonette JA, Clevenger AP, Cutshall CD, et al.. (2003) Road Ecology: Science and Solutions. Washington, DC, USA. Island Press. 481 p.

[4] Gordon IJ (2009) What is the future for wild, large herbivores in human-modified agricultural landscapes. Wildl Biol 15: 1–9.

[5] Colino–Rabanal VJ (2011) Contribuciones al análisis de mortalidad de vertebrados en carreteras. PhD Thesis. Facultad de Ciencias Agrarias y Ambientales. Salamanca. Universidad de Salamanca.

[6] Conover MR (2002) Resolving wildlife conflicts: the science of wildlife damage management. Lewis, Boca Raton, Florida, USA. CRC Press. 440 p.

[7] Malo JE, Suárez F, Díez A (2004) Can we mitigate animal-vehicle accidents using predictive models? J Applied Ecol 41: 701–710.

[8] Seiler A (2003) The toll of the automobile: wildlife and roads in Sweeden. PhD Thesis. Uppsala. Swedish University of Agricultural Sciences.

[9] Gunson KE, Mountrakis G, Quackenbush LJ (2011) Spatial wildlife-vehicle collision models: A review of current work and its application to transportation mitigation projects. J Environ Manage 92: 1074–1082. 21190788 10.1016/j.jenvman.2010.11.027

[10] Ascensao F, Clevenger A, Santos-Reis M, Urbano P, Jackson N (2013) Wildlife-vehicle collision mitigation: is partial fencing the answer? An agent-based model approach. Ecol Model 257: 36–43.

[11] Ng JW, Nielsen C, St. Clair CC (2008) Landscape and traffic factors influencing deer-vehicle collisions in an urban environment. Human-Wildlife Conflicts 2: 34–47.

[12] Clevenger AP, Chruszcz B, Gunson KE (2001) Highway mitigation fencing reduces wildlife–vehicle collisions. Wildlife Soc Bul 29: 646–653.

[13] Álvarez J, Bea A, Faus JM, Castién E, Mendiola I (1985) Atlas de los vertebrados continentales de Álava, Vizcaya y Guipúzcoa. Bilbao. Gobierno Vasco. 336 p.

[14] Ministerio de Medio Ambiente y Medio Rural Marino (2008) Prescripciones técnicas para el seguimiento y evaluación de la efectividad de las medidas correctoras del efecto barrera de las infraestructuras de transporte. Documentos para la reducción de la fragmentación de hábitats causada por infraestructuras de transportes, número 2. Madrid. O.A. Parques Nacionales. Ministerio de Medio Ambiente y Medio Rural y Marino. 115 p.

[15] Aihartza J, Zuberogoitia I, Camacho E, Torres JJ (1999) Status of carnivores in Biscay (N Iberian peninsula). Miscelanea Zoologica 22: 41–52.

[16] Palazón S, Melero Y, Gómez A, López de Luzuriaga J, Podra M, et al (2012) Causes and patterns of human-induced mortality in the Critically Endangered European mink *Mustela lutreola* in Spain. Oryx 46: 614–616.

[17] Acevedo P, Vicente J, Alzaga V, Cortázar C (2009) Wild Boar abundance and hunting effectiveness in Atlantic Spain: environmental constraints. Galemys 21(2): 13–29.

[18] Palomo LJ, Gisbert J (2002) Atlas de los mamíferos terrestres de España. Madrid. Dirección General de Conservación de la Naturaleza-SECEM-SECEMU. 564 p.

[19] Monge J (2012) La caza en el País Vasco. Foresta 55: 54–60.

[20] Instituto Nacional de Estadistica (Spanish Statistical Office) (2014) Demografía y Población. Available: http://www.ine.es/. Accessed 2014 May 7.

[21] Emap (2011) Evolución del tráfico en las carreteras de Bizkaia. Bilbao. Departamento de Obras Públicas y Transportes. 547 p.

[22] Zuberogoitia I, Castillo I, Zabala J, Iraeta A, Azkona A (2011) Population trends of diurnal forest raptors in Biscay. In Zuberogoitia I, Martínez JE, editors. Ecology and Conservation of European Forest-dwelling Raptors. Bilbao. Diputación Foral de Bizkaia. Pp 70–80.

[23] GeoEuskadi (2013) Índice de Cartografía. Cartografía básica. Ortofotos. Orto 2011. Available: ftp://ftp.geo.euskadi.net/cartografia/Cartografia_Basica/Ortofotos/ORTO_2011/. Accessed 2013 Sept 30.

[24] Diputación Foral de Bizkaia (2014) Departamento de Obras Públicas y Transportes. Evolución del Tráfico. Available: http://www.bizkaia.net/. Accessed 2014 May 7.

[25] Google Earth Street View (2013) Available: http://maps.google.es/support/bin/answer/. Accessed 2013 Sept 30.

[26] Hothorn T, Brandl R, Müller J (2012) Large-Scale Model-Based Assessment of Deer-Vehicle Collision Risk. PLoS ONE 7(2): e29510 doi:10.1371/journal.pone.0029510 22359535 10.1371/journal.pone.0029510 PMC3281017

[27] Zar JH (1999) Biostatistcal analysis. New Jersey: Prentice Hall. 663 p.

[28] Zuberogoitia I, González-Oreja JA, Martínez JE, Zabala J, Gómez I, et al (2013) Foraging movements of Eurasian Griffon Vultures (*Gyps fulvus*): implications for supplementary feeding management. European J Wildl Res 59: 421–429.

[29] López-López P, Zuberogoitia I, Alcantara M, Gil JA (2013) Philopatry, natal dispersal, first settlement and age of first breeding of Bearded Vultures *Gypaetus barbatus* in central Pyrenees. Bird Study 60: 550–560.

[30] Hood GM (2013) Poptools. Available: http://www.poptools.org. Accessed 2013 Sept 30.

[31] Serrano D, Carrete M, Tella JL (2008) Describing dispersal under habitat constraints: A randomization approach in lesser kestrels. Basic Appl Ecol 9: 771–778.

[32] Burnham KP, Anderson DR (2002) Model selection and multi-model inference: a practical information-theoretic approach. Berlin. Springer. 488 p.

[33] Wagenmakers EJ, Farrel S (2004) AIC model selection using Akaike weights. Psychonomic Bulletin & Review 11: 192–196. 15117008 10.3758/bf03206482

[34] Wahlström LK, Liberg O (1995) Patterns of dispersal and seasonal migration in Roe Deer (Capreolus capreolus). J Zool 235: 455–467.

[35] Coulon A, Cosson JF, Angibault JM, Cargnelutti B, Galan M, et al (2004) Landscape connectivity influences gene flow in a Roe Deer population inhabiting a fragmented landscape: an individual-based approach. Mol Ecol 13: 2841–2850. 15315694 10.1111/j.1365-294X.2004.02253.x

[36] Madsen AB, Strandgaard H, Prang A (2002) Factors causing traffic killings of Roe Deer *Capreolus capreolus* in Denmark. Wildl Biol 8: 55–61.

[37] Saïd S, Gaillard JM, Duncan P, Guillon N, Guillon N, et al (2005) Ecological correlates of home-range size in spring-summer for female Roe Deer (*Caprimulgus europaeus*) in a deciduous woodland. J Zool Lond 267: 301–308.

[38] Saïd S, Servanty S (2005) The influence of landscape structure of female Roe Deer home-range size. Landscape Ecol 20: 1003–1012.

[39] Balčiauskas L, Balčiauskien·e L (2008) Wildlife-vehicle accidents in Lithuania, 2002–2007. Acta Biol Univ Daugavp 8: 89–94.

[40] Peris S, Baquedano R, Sánchez A, Pescador M (2005) Mortalidad del jabalí (Sus scrofa) en carreteras de la provincia de Salamanca (NO de España): ¿influencia de su comportamiento social? Galemys 17: 13–23.

[41] Díaz ER, Marey MF, Vázquez I, Álvarez CJ (2010). Análisis espacial de las colisiones de vehículos con animales silvestres en la red vairia de la provincia de Lugo (España). Madrid. XIV International Congrees on Project Engineering 1196–1207.

[42] Scillitani L, Monaco A, Toso S (2010) Do intensive drive hunts affect Wild Boar (*Sus scrofa*) spatial behaviour in Italy? Some evidences and management implications. Eur. J Wildl Res 56: 307–318.

[43] Thurfjell H (2011) Spatial Behaviour of Wild Boar. PhD Thesis. Umea. Swedish University of Agricultural Sciences.

[44] Ford AT, Clevenger AP, Huijser MP, Dibb A (2011) Planning and prioritization strategies for phased highway mitigation using wildlife-vehicle collision data. Wildl Biol 17: 253–265.

[45] VerCauteren KC, Lavelle MJ, Hygnstrom S (2006) Fences and deer-damage management: a review of design and efficacy. Wildlife Soc Bul 34: 191–200.

[46] Found R, Boyce MS (2011) Predicting deer-vehicle collisions in an urban area. J Environ Manage 92: 2486–2493. 21700381 10.1016/j.jenvman.2011.05.010

[47] Rodríguez-Refojos C, Zuberogoitia I (2011) Middle-sized carnivores in mosaic landscapes: the case of Biscay (Sw Europe). In Rosalino LM, Gheler Costa C, editors. Middle-sized carnivores in agricultural landscapes. New York. Nova Science Publishers. Pp. 105–126.

[48] Torres RT, Carvalho JC, Panzaccho M, Linnell JDC, Fonseca C (2011) Comparative use of forest habitats by Roe Deer and moose in a human-modified landscape in southeastern Norway during winter. Ecol Res 26: 781–789.

[49] Fonseca C (2008) Winter habitat selection by Wild Boar *Sus scrofa* in south eastern Poland. Eur J Wildl Res 54: 361–366.

[50] Colino–Rabanal VJ, Bosch J, Muñoz MJ, Peris SJ (2012) Influence of new irrigated croplands on Wild Boar (*Sus scrofa*) road kills in NW Spain. Anim Biodivers Conserv 35: 247–252.

[51] Gurrutxaga M, Lozano PJ, del Barrio G (2010) GIS-based approach for incorporating the connectivity of ecological networks into regional planning. J Nat Conserv 18: 318–326.

[52] Zuberogoitia I, Zalewska H, Zabala J, Zalewski A (2013) The impact of river fragmentation on the population persistence of native and alien mink: an ecological trap for the endangered European mink. Biodiv Conserv 22: 169–186.

[53] Huijser MP, Duffield JW, Clevenger AP, Ament RJ, McGowen PT (2009) Cost-benefit analyses of mitigation measures aimed at reducing collisions with large ungulates in the United States and Canada: a decision support tool. Ecology and Society 14(2): 15 (online). Available: http://www.ecologyandsociety.org/voll4iss2/art15/. Accessed 2013 Dec 20.

